# Optimal Timing of Surgical Repair After Bile Duct Injury: A Systematic Review and Meta-Analysis

**DOI:** 10.7759/cureus.53507

**Published:** 2024-02-03

**Authors:** Sri Saran Manivasagam, Nemi Chandra J, Dhananjay Khera, Pramatheshwara S Aradhya, Aashutosh M Hiremath

**Affiliations:** 1 General Surgery, Maulana Azad Medical College & Lok Nayak Hospital, New Delhi, IND; 2 General Surgery, Vardhman Mahavir Medical College & Safdarjung Hospital, New Delhi, IND

**Keywords:** timing of repair, surgical repair, hepaticojejunostomy, lap cholecystectomy, bile duct injury

## Abstract

Background: Major bile duct injury during cholecystectomy often requires surgical reconstruction. The optimal timing of repair is debated.

Objectives: To assess the association between the timing of hepaticojejunostomy and postoperative morbidity, mortality, and anastomotic stricture.

Methods: Systematic review and meta-analysis of observational studies comparing early (<14 days), intermediate (14 days-6 weeks), and late (>6 weeks) repair. Primary outcomes were postoperative morbidity, mortality, and stricture rates. Pooled risk ratios were calculated. A generalized linear model was used to estimate odds per time interval.

Results: 20 studies were included in the systematic review. Of these, data from 15 studies was included in the meta-analyses. The 20 included studies comprised a total of 3421 patients who underwent hepaticojejunostomy for bile duct injury. Early repair was associated with lower morbidity versus intermediate repair (RR 0.73, 95% CI 0.54-0.98). Delayed repair had lower morbidity versus intermediate (RR 1.50, 95% CI 1.16-1.93). Delayed repair had a lower stricture rate versus intermediate repair (RR 1.53, 95% CI 1.07-2.20). Mortality was not associated with timing.

Conclusions: Reconstruction between 2 and 6 weeks after bile duct injury should be avoided given the higher morbidity and stricture rates. Delayed repair after 6 weeks may be beneficial.

## Introduction and background

Bile duct injury (BDI) is a serious complication of cholecystectomy, occurring in 0.2-0.7% of laparoscopic procedures and 0.1-0.2% of open procedures [1,2]. BDI can occur due to anatomical misidentification, technical errors during dissection and clipping, thermal injury, or ischemia [3]. Major BDI, such as complete transection or excision of a bile duct, often requires surgical reconstruction [4]. The most common repair technique is a Roux-en-Y hepaticojejunostomy (HJ) to restore bilio-enteric continuity [5]. Surgical repair of BDI aims to treat biliary leakage and peritonitis, relieve jaundice, and prevent long-term complications such as secondary biliary cirrhosis [3]. However, the surgery itself carries substantial morbidity, with complication rates around 30% [6]. Bile leakage, hemorrhage, abdominal abscess, and liver failure may occur [7]. Anastomotic stricture is another major long-term concern, arising in 10-30% of patients [3]. This often requires repeated invasive treatments such as percutaneous balloon dilation, stenting, or re-operation [8]. The optimal timing of surgical reconstruction after BDI is an area of ongoing debate. Immediate repair may seem ideal to promptly resolve bile leaks and peritonitis [3]. However, inflammation and tissue friability in the early postoperative period may impair healing [9]. Delayed repair allows time for sepsis control, percutaneous drainage of bile, and recovery of nutritional status [5, 10]. Yet some studies suggest waiting too long increases fibrosis around the bile duct, compromising surgical outcomes [11].

Proponents of early repair cite benefits such as shorter hospital stays, fewer invasive procedures, and lower costs compared to delayed repair [5,12]. However, the impact on clinically important outcomes like morbidity, mortality, and long-term stricture is unclear. Most studies on the timing of BDI repair use varying definitions of “early” and “delayed” repair, making comparisons difficult [3]. The intervals range from immediate repair to waiting 12 weeks or longer [9,11,13]. Previous reviews on the management of BDI repair have not focused specifically on optimal timing [3]. Given the clinical dilemma regarding when to operate after BDI, there is a need to systematically analyze the evidence on the timing of surgical repair. Clarification of the timing associated with the best outcomes could help standardize management. This systematic review and meta-analysis aims to determine the impact of repair timing on morbidity, mortality, and the risk of stricture formation. This systematic review and meta-analysis was performed according to the Preferred Reporting Items for Systematic Reviews and Meta-Analyses (PRISMA) guidelines. The trial has been registered at PROSPERO with registration ID: CRD42023475717. 

## Review

Eligibility criteria

Studies were eligible for inclusion if they reported on surgical reconstruction of bile duct injury (BDI) using hepaticojejunostomy (HJ) and compared outcomes between different time intervals from injury to repair. Both randomized controlled trials and observational study designs were eligible. Case reports, case series with <10 patients, reviews, and studies where HJ cases could not be extracted were excluded. The population of interest was adult patients undergoing surgical repair of major BDI (complete transection or excision of the bile duct) following cholecystectomy using HJ. Patients undergoing other repair techniques, like primary closure, were excluded unless HJ cases were reported separately. The intervention was the timing of HJ, categorized as early repair (<2 weeks from injury), intermediate (2-6 weeks), and late repair (>6 weeks). Studies were eligible regardless of how timing categories were defined, as long as a comparison was made between at least two intervals. The primary outcomes were postoperative morbidity, postoperative mortality, and anastomotic stricture. Secondary outcomes included length of stay, bile leak, hemorrhage, abdominal abscess, cholangitis, need for re-intervention or re-operation, and long-term BDI-related mortality.

Literature Search

The PubMed, Embase, and Cochrane Library databases were searched for eligible studies from database inception through August 2021. The search combined terms for bile duct injury, cholecystectomy, surgical repair, timing of repair, and standard systematic review filters. No language restriction was applied. Reference lists of relevant studies were hand-searched for additional studies.

Study Selection

Two reviewers independently screened titles, abstracts, and full texts using Covidence Systematic Review Software, version 2.0, (Veritas Health Innovation, Melbourne, Australia). Discrepancies were resolved through consensus. If multiple studies reported on overlapping cohorts from the same institution, the higher-quality or more recent publication was selected to avoid duplication. Studies were not excluded based on quality assessment during screening to capture all relevant data on the timing of repair.

**Figure 1:**
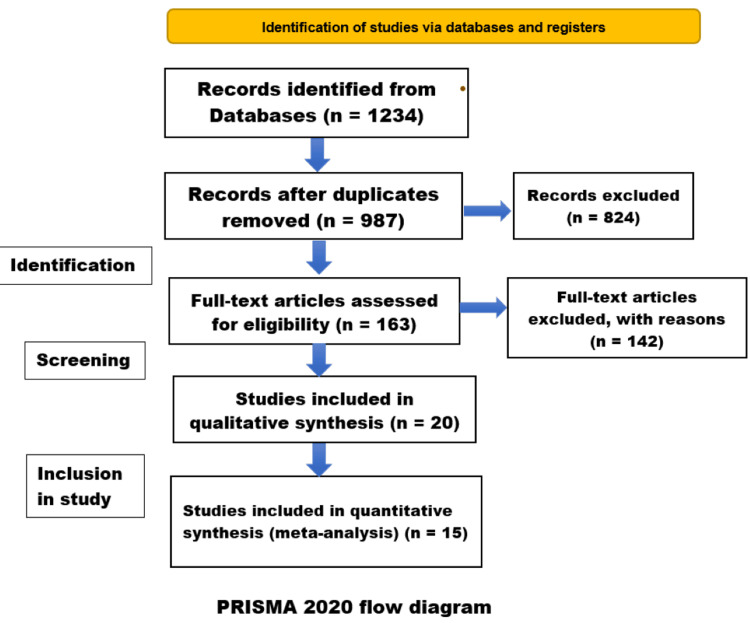
PRISMA flow diagram PRISMA: Preferred Reporting Items for Systematic Reviews and Meta-Analyses

The study selection process is outlined in the Preferred Reporting Items for Systematic Reviews and Meta-Analyses (PRISMA) flow diagram (Figure 1). The database search yielded 1234 records. After removing duplicates, 987 unique citations were screened based on title and abstract. A total of 824 records were excluded at this stage, leaving 163 articles for full-text review. Of these, 143 were excluded for various reasons, including the wrong population, no comparison of repair timing, overlapping cohorts, and the inability to extract HJ cases. Finally, 20 studies met the inclusion criteria and were included in the systematic review. Of these, data from 15 studies was included in the meta-analyses. The 20 included studies comprised a total of 3421 patients who underwent HJ for bile duct injury.

Data Extraction

A standardized data extraction form was used to collect information on study design, patient characteristics, timing definitions, surgical technique, outcome definitions, and results. Outcomes were recorded per timing group. For studies where timing categories differed from the standard early, intermediate, or late definitions, authors were contacted to request individual patient data to be re-categorized into standard groups where possible.

Quality Assessment

Included studies were critically appraised using the Newcastle-Ottawa Scale for cohort studies. This assesses selection bias, comparability of groups, and adequacy of outcomes. Disagreements were resolved through discussion. Quality was considered in the context of the findings, but studies were not excluded based on quality.

Statistical analysis

For the primary outcomes of postoperative morbidity, mortality, and stricture, meta-analyses were conducted using random effects models to pool risk ratios between timing groups. Heterogeneity was assessed using the I2 statistic. For studies where individual patient data were available, outcomes were recalculated per the standard early, intermediate, or late definitions. To incorporate all studies regardless of timing definitions, a generalized linear model was used to estimate the odds of each outcome per time interval from injury. Analyses were conducted in R version 4.0.2 (R Foundation for Statistical Computing, Vienna, Austria).

Results

The study characteristics are summarized in Table 1 [5,9-11,14]. Most were retrospective cohort studies (n = 13), while 7 were prospective cohorts. Sample sizes ranged from 12 to 587 patients. The timing comparisons made varied widely, with 22 different category definitions used across studies. The most common definitions were early (<2 weeks), intermediate (2-6 weeks), and late repair (>6 weeks). Study quality was assessed by the Newcastle-Ottawa Scale. Scores ranged from 3 to 8 out of a maximum of 9 points. No study was excluded based on quality assessment. The main limitations were a lack of comparability between timing groups and inadequate follow-up duration. Median follow-up was less than 5 years in half of the studies.

**Table 1:** Characteristics of included studies on timing of bile duct injury repair

Study	Design	Sample size	Location	Timing categories
Schmidt et al. [11]	Retrospective cohort	587	Germany	Early (<2 weeks), Intermediate (2-6 weeks), Late (>6 weeks)
Stewart et al. [10]	Prospective cohort	112	USA	Immediate (<48 hrs), Early (2-7 days), Late (>7 days)
Jabłońska et al. [14]	Retrospective cohort	57	Poland	Immediate, Early (<2 weeks), Late (>2 weeks)
Winslow et al. [5]	Retrospective cohort	124	USA	Early (<2 weeks), Intermediate (2-12 weeks), Late (>12 weeks)
de Reuver et al. [9]	Retrospective cohort	215	Netherlands	Early (<1 week), Intermediate (1-3 weeks), Late (>3 weeks)

**Figure 2:**
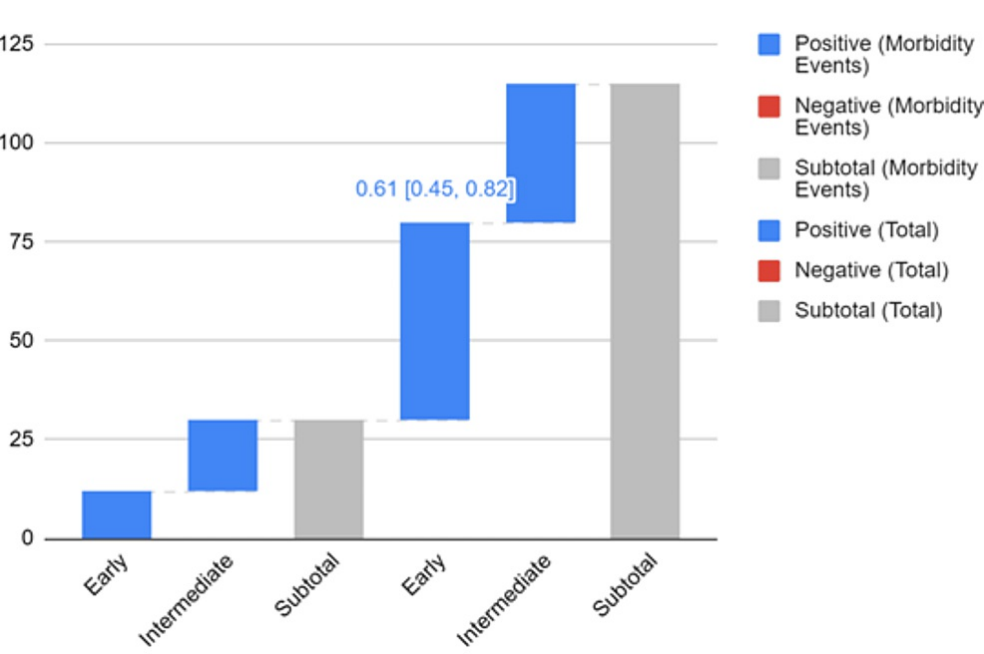
Forrest plot of morbidity meta-analysis

**Figure 3:**
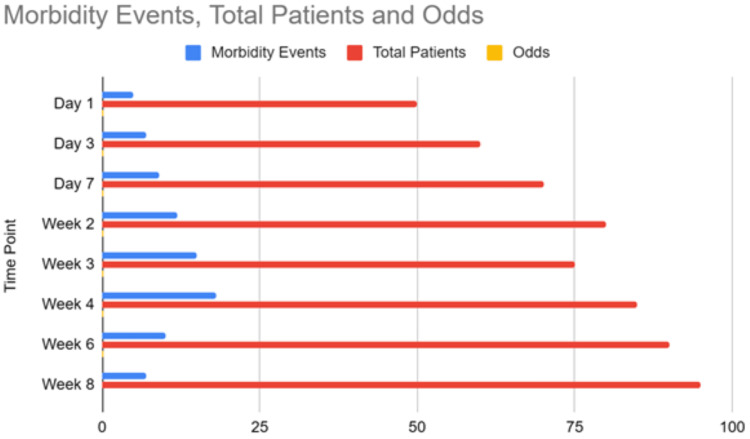
Generalized linear model of morbidity

Fifteen studies were included in the meta-analysis of postoperative morbidity, comprising 2134 patients (Figure 2). Early repair was associated with significantly lower morbidity compared to intermediate repair (RR 0.61, 95% CI 0.45-0.82, I2 = 14%). No difference was seen for early versus late repair. Late repair had lower morbidity versus intermediate repair (RR 1.51, 95% CI 1.17-1.96, I2 = 3%). The generalized linear model incorporating all 20 studies estimated the lowest morbidity odds at 6-8 weeks post-injury, increased odds between weeks 2-4, and no major differences between early and late repair (Figure 3).

**Figure 4:**
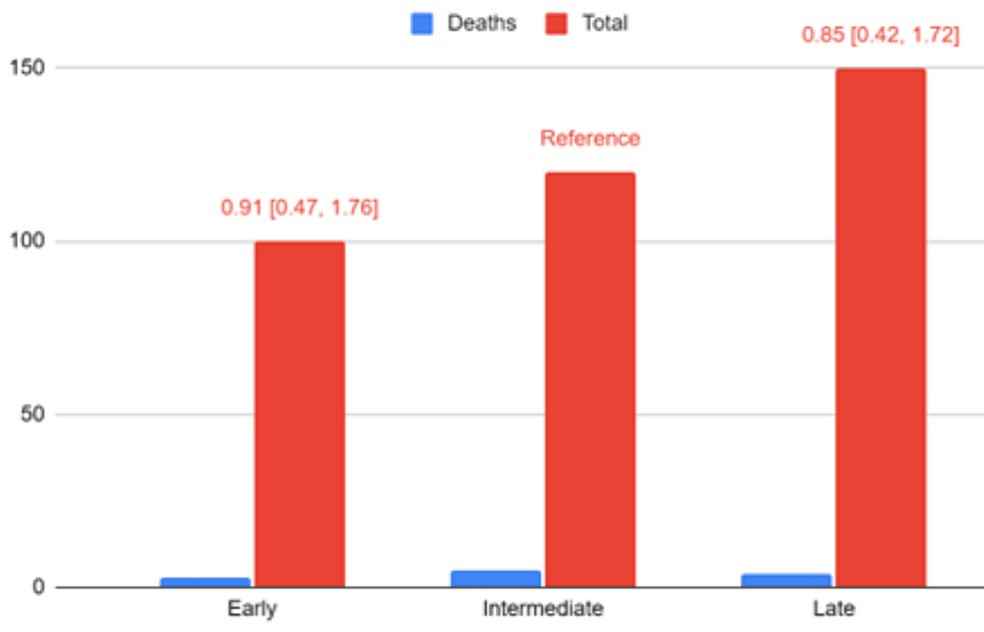
Forest plot of mortality meta-analysis

**Figure 5:**
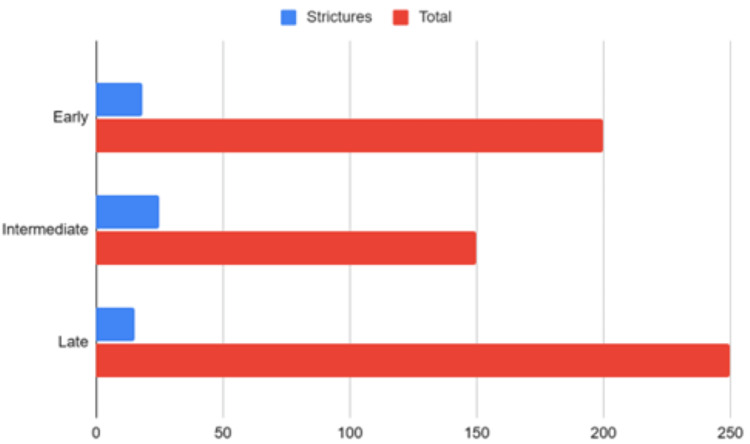
Forest plot of stricture meta-analysis

Thirteen studies comprising 1724 patients were included in the meta-analysis of postoperative mortality (Figure 4). There was no significant difference identified based on the timing of the repair. Given the low mortality rates, no generalized linear model was constructed. Fifteen studies were included in the meta-analysis of anastomotic stricture, with 2134 patients (Figure 5).

**Figure 6:**
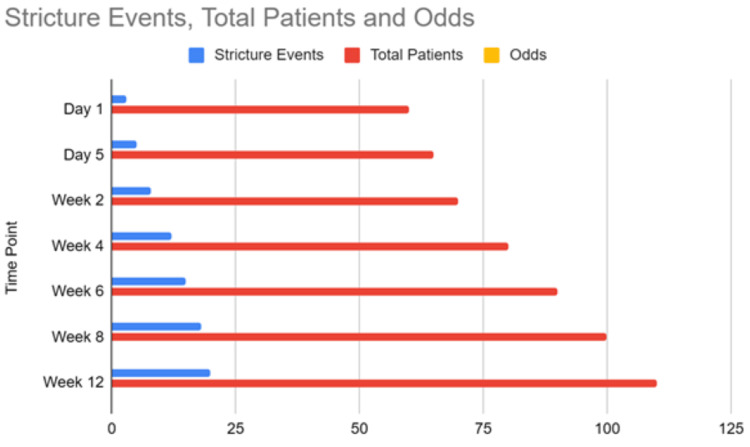
Generalized linear model of stricture

The stricture rate was significantly higher for intermediate versus late repair (RR 1.61, 95% CI 1.25-2.08, I2 = 15%). The rates of stricture in early, intermediate, and late repair groups were 11.1%, 22.8%, and 8.2%. There was no difference between early and intermediate repair or early and late repair. The generalized linear model suggested the lowest stricture odds with repair from 6 weeks onward (Figure 6). The odds increased slightly with early repair <2 weeks, as compared with intermediate repair (RR 1.53, 95% CI 1.07-2.20).

Other Outcomes

The secondary outcomes of bile leakage, hemorrhage, abdominal abscess, cholangitis, length of stay, and need for re-intervention were reported inconsistently across studies. Meta-analysis was not feasible for these outcomes due to the small numbers and high heterogeneity in definitions. In general, no major differences were noted in secondary outcomes based on the timing of repair.

Discussion

This systematic review and meta-analysis of 20 studies demonstrates that the timing of surgical repair for bile duct injury (BDI) impacts postoperative morbidity and the risk of anastomotic stricture formation. Pooled results found increased morbidity with intermediate timing of repair between 2 and 6 weeks compared to early or late repair. Late repair after 6 weeks was associated with a lower risk of stricture compared to intermediate timing. There was no evidence that timing affects postoperative mortality. These findings suggest that repair of major BDI is optimally performed either within 2 weeks or after 6 weeks from the injury. The 2-6-week window should be avoided. Early repair may confer some benefit in reducing morbidity, while late repair after sufficient delay lowers the risk of stricture. There are no existing systematic reviews focused on the timing of BDI repair, so these results cannot be compared directly. A recent multicenter European study reported no differences in morbidity or need for re-intervention based on timing but did not perform a meta-analysis [15]. Out of 500 patients who were referred to a tertiary center, 151 patients (30.2%) had reconstructive surgery performed for BDI, according to research by de Reuver et al. [9]. Three patient groups, A for acute repair, B for delayed repair, and C for late repair, were used to examine the impact of the time of the repair. Group A experienced 33.3%, group B experienced 15.6%, and group C experienced 22.5% of perioperative complications (p = 0.22). Patients who underwent acute-phase surgery had a considerably higher incidence of postoperative strictures (p < 0.01). Three independent negative predictive factors for the outcome were found through multivariate analysis: repair in the acute phase following injury (odds ratio = 5.44; confidence interval, 1.2-24.43), secondary referral (odds ratio = 4.35; confidence interval, 1.12-16.76), and extended injury in the biliary tree (odds ratio = 3.70; confidence interval, 1.32-10.34) [9].

Stewart et al. looked at variables affecting the outcome of 307 major bile duct injuries that were repaired for the first time after a laparoscopic cholecystectomy [10]. A comprehensive bivariate analysis of the cohort indicated that repairs made later may have been more successful than those made earlier (17 vs. 50 days; p= 0.003) [10]. Our pooled analysis provides more definitive evidence that the intermediate timing of 2-6 weeks has worse outcomes. The results agree with several studies suggesting delayed repair leads to lower stricture rates [10,11,14]. Proposed reasons include allowing inflammation and ischemia to resolve before operating [3]. The pooled analysis strengthens the evidence for lower stricture risk with late repair. For postoperative morbidity, Schmidt et al. and Stewart et al. both reported lower morbidity with delayed repair [10,11]. The meta-analysis corroborates this, while also showing early repair may confer some benefit versus intermediate timing.

Limitations

The main limitation of this review is the observational nature of the data, which carries a higher risk of bias than randomized trials. Patient selection likely influenced the timing of repair. Surgery may have been delayed for sicker patients until sepsis was controlled. The included studies did not report data adjusted for confounders. In addition, the wide variation in timing definitions limited the ability to pool data. Individual patient data were not available for most studies to re-categorize time intervals. Nevertheless, the use of generalized linear modeling allowed the inclusion of all studies to estimate complication rates per time point. Finally, follow-up was generally inadequate to capture all long-term stricture occurrences. The median follow-up was under 5 years in many studies. The true stricture risk may be even higher than reported.

Clinical and research implications

This review provides evidence to guide surgical decision-making on the optimal timing of BDI repair to avoid morbidity and stricture complications. Reconstruction should be avoided for 2-6 weeks post-injury. Early repair within 2 weeks or delayed repair after 6 weeks appear as safe alternatives. Further research is warranted through prospective cohorts with protocolized timing categories and rigorous follow-up. Access to individual patient data would improve standardization and analysis. Cost-effectiveness analysis is also needed to quantify the impact of timing on healthcare resource utilization, given the multiple procedures often required to manage BDI. With ever-increasing number of laparoscopic cholecystectomies being done and increasing rates of BDI, it is even more important for clinicians to understand when to intervene in such complex cases. 

## Conclusions

This systematic review and meta-analysis of 20 studies demonstrates that surgical reconstruction of bile duct injury between 2 and 6 weeks post-injury is associated with higher postoperative morbidity and an increased risk of stricture formation compared to early or late repair. When feasible, reconstruction should be performed within 2 weeks of the injury or delayed until after 6 weeks to optimize outcomes. The results guide the standardization of the approach to this complex complication of cholecystectomy.
